# Developing Palliative Medicine as an Accredited Medical Specialty in Kenya

**DOI:** 10.1200/GO.22.00025

**Published:** 2022-05-20

**Authors:** Hussein Elias, Lindsay A. Dow, Juli Boit, Chite F. Asirwa, Kenneth Cornetta

**Affiliations:** ^1^ Department of Family Medicine, Moi University School of Medicine, Eldoret, Kenya; ^2^ Icahn School of Medicine at Mount Sinai, New York, NY; ^3^ Living Room International, Eldoret, Kenya; ^4^ International Cancer Institute, Eldoret, Kenya; ^5^ Department of Medical and Molecular Genetics, Indiana University School of Medicine, Indianapolis, IN

## Abstract

Most people living with life-limiting illnesses in Kenya lack access to palliative care. Globally, palliative medicine is a growing specialty that equips clinicians with the training required to improve the quality of life for people living with a wide variety of serious illnesses. Optimal delivery relies on a skilled workforce with specialty-level training, and we identified the absence of board-accredited training programs for clinical officers (COs) and physicians as a barrier to providing high-quality palliative care in Kenya.

**METHODS:**

We held a series of stakeholder meetings with expert palliative care clinicians, leaders, and educators from Kenya and other countries to develop and implement a comprehensive, evidence-based palliative medicine curriculum for COs.

**RESULTS:**

We developed a higher diploma program that is being administered by the Moi Teaching and Referral Hospital College in Eldoret, Kenya, with faculty from Moi University School of Medicine and affiliated institutions. We have collaborated to create the first diploma awarding program in palliative medicine in Kenya. Our efforts have led the Kenyan CO Council adding palliative medicine to their list of recognized and licensed specialties. COs are now enrolled in an 18-month program that will lead to a higher diploma and national recognition as palliative care specialists.

**CONCLUSION:**

Early building of consensus and educating policymakers, regulatory bodies, and government personnel was an important step to overcome the challenge of palliative care misconceptions. The unique capacity of global partnerships and early and frequent stakeholder involvement is critical in novel program development. Local ownership of such in-country programs is key, and the stakeholders should be included in strategies for sustainability.

## INTRODUCTION

Palliative care is defined by the WHO as “an approach that improves the quality of life of patients and their families who are facing problems associated with life-threatening illness through early identification, correct assessment and treatment of pain and other problems, whether physical, psychosocial and spiritual.”^1^ Although there is consensus that relief of pain and suffering is an ethical duty of health care professionals and health care systems,^1-3^ each year approximately 40 million people are in need of palliative care, of whom 78% live in low- and middle-income countries.^1-3^ Furthermore, there is an increasing need of palliative care services given the rapidly increasing number of patients with noncommunicable diseases and the aging of the population.^2,4^

**CONTEXT**

**Key Objective**
There is an absence of board-accredited palliative and hospice care specialty training in Kenya. This article describes the key processes in developing an accredited specialty training program.
**Knowledge Generated**
Early building of consensus and educating policymakers, regulatory bodies, and government personnel is critical in novel program development. In addition, early and frequent stakeholder involvement and the unique capacity of global partnership also play a key role.
**Relevance**
We have learned that the development of a robust and sustainable novel specialty training program requires early involvement of a diverse group of stakeholders with expertise in leadership and education. The training must also include a variety of clinical competencies tailored to the specialty training in the local context.

Access to palliative care is limited in most African countries.^2,5^ In Kenya, access is concentrated in urban areas.^4^ A study in 2018 found only 3% of health facilities offer palliative care services of which only 5% had morphine available.^6^ Through the Kenya Hospice and Palliative Care Association, in partnership with the Ministry of Health, efforts are underway to integrate palliative care into medical and nursing training programs as well as government health facilities.^5,6^ While there is progress, implementation has encountered significant challenges related to the limited number of hours allocated, inadequate palliative care teaching staff, and inadequate knowledge in palliative care among the staff in clinical areas.^7^ These data speak to a limited workforce for providing clinical palliative care and for educating health care workers. Currently, the specialty of palliative medicine is recognized by Kenya Medical Board, but there are no residencies or fellowships in the country. A higher diploma program for nurse practitioners is recognized by the Nursing Council, but the Kenyan Clinical Officer (CO) Council did not recognize palliative care as a specialty. Given the need to improve services, we explored the development of training programs to expand the palliative care workforce.

## METHODS

### Curriculum Development and Accreditation

#### Needs Assessment.

An initial assessment was performed that considered the number of people in need of palliative care, the availability of structural and organizational resources, and the existing workforce in Kenya. The assessment was done in two ways: (1) literature search focused on current gaps in the provision of equitable palliative care and (2) stakeholder meetings with representatives from Moi University School of Medicine, Moi Teaching and Referral Hospital (MTRH) College, health care regulatory bodies, oncologists, and family medicine and palliative medicine physicians, along with practicing palliative care clinicians representing nursing and CO viewpoints.

Our survey of palliative care training in Kenya noted several domestic and foreign online training programs, but none were recognized as degree/higher diploma granting of board specialty programs. Our review of existing literature found that the WHO and many other organizations stress the critical need for improved education. The WHO publication “Integrating palliative care and symptom relief into primary health care: a WHO guide for planners, implementers and managers”^3^ and a recent WHO publication on quality health services and palliative care^8^ provide a detailed discussion of needed services but do not provide specific educational objectives for palliative care specialists.

We identified the limited number of palliative care specialists as a significant challenge to developing domestic training programs since qualified educators are vital to obtaining national certification for a new program. Recognizing this limitation, the existing collaboration between Moi University and North American Medical Schools (AMPATH) became an integral part of the curriculum development effort. AMPATH Palliative Care has a 20-year relationship with Moi, and before our stakeholder meetings, North American colleagues shared fellowship curricula. We presented the following points which we felt were influential in developing a training program: (1) delivery of palliative care requires a multidisciplinary interprofessional team and program graduates should foster a collaborative care model that addresses physical, psychosocial, and spiritual distress; (2) there is a growing movement to incorporate palliative care upstream at the time of diagnosis of life-limiting illnesses, especially for people with high symptom burden or distress; (3) individuals with life-limiting illness have a broad spectrum of symptoms and the scope of practice is much more than pain control; (4) symptom management, particularly with opioid medications, must be tailored to each individual and this skill is best developed through mentored clinical practice; (5) while cancer incidence is rising in low- and middle-income countries, so are other noncommunicable diseases such as heart disease, lung disease, kidney disease, stroke, and dementia which should be included in the curriculum; (6) prognostication is an essential part of palliative medicine, and therefore, a broad exposure to common life-limiting illnesses is needed; and (7) communication and counseling around prognosis and illness trajectory is a learned skill and best developed through mentored learning.

#### Stakeholder Involvement.

The first draft of the curriculum was a certificate-level training program for all clinicians at the primary level of care, including medical officers, COs, and nurses. Unlike other certificate programs, the 1-year training program predominantly mentored clinical practice within inpatient and outpatient hospital and hospice rotations. During the first stakeholder meeting, two main recommendations were provided: The first was to develop separate training programs for each discipline of clinicians, and the second was that the didactic contact and mentored clinical exposure of clinicians may qualify for a higher diploma degree which regulatory bodies could recognize as a subspecialty training in palliative care.

On the basis of these recommendations, the curriculum was revised as a subspecialty higher diploma training for COs to be housed in the MTRH College of Health Sciences. COs were selected as the first program given their ubiquitous position throughout the Kenyan Health Care system. COs (also known as nonphysician clinicians) are middle-level professionals with less training and a more limited scope of practice.^9^ The ratio of COs to medical doctors in 2015 was 2.7 to 1.5 per 10,000 population.^10^ The COs' council accredits general COs and specialist COs.^9^ Having a representative from the Kenyan CO Council improved our curriculum and stimulated discussion within the Council that led to the recognition of palliative medicine as a specialty.

A total of five subsequent stakeholder meetings were conducted over a 3-year period (delayed in part by COVID-19) to finalize content and meet the requirements of the MTRH College and the CO regulatory body. This process was guided by the faculty of Moi University School of Medicine, MTRH clinicians, and MTRH College Directors who are ultimately responsible for the content and staffing of the training program. The selection and invitation of the stakeholders was based on the following objectives: the need of broad input from educators in designing a competency-based training, content experts in palliative medicine to develop the core content, national representation to meet accreditation requirements, and input from practicing clinicians. Participating stakeholders included palliative care specialists (African and North American), curriculum development experts, advocacy representation (Kenya Hospice and Palliative Care Association), representatives of national accrediting bodies (Kenya CO Council and Nursing Council of Kenya), and MTRH College administration (director, deputy director academics, and member of curriculum committee). Stakeholders also included practicing COs, nurses, oncologists, and family physicians. Educators from other higher diploma programs (eg, oncology, chronic disease care, and child health) also provided practical advice on program design. The learning objectives for the 18-month training are provided in Table 1. The curriculum organization is provided in Table 2, and the schedule of weekly activities is shown in Table 3. Students will rotate through medical, surgical, and pediatrics wards, as well as specialty clinics at a national referral hospital, the palliative care service at a county (district) referral hospital, and the inpatient and outpatient services at a hospice center.

**TABLE 1:**
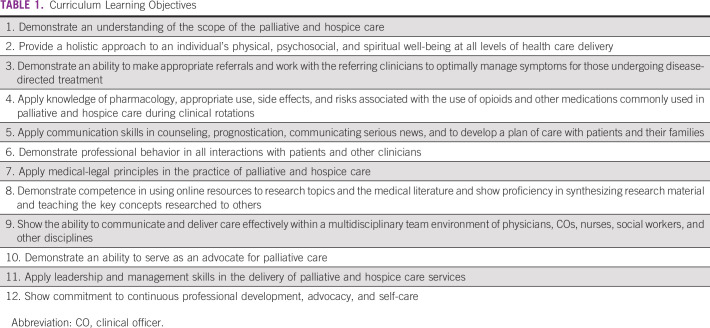
Curriculum Learning Objectives

**TABLE 2:**
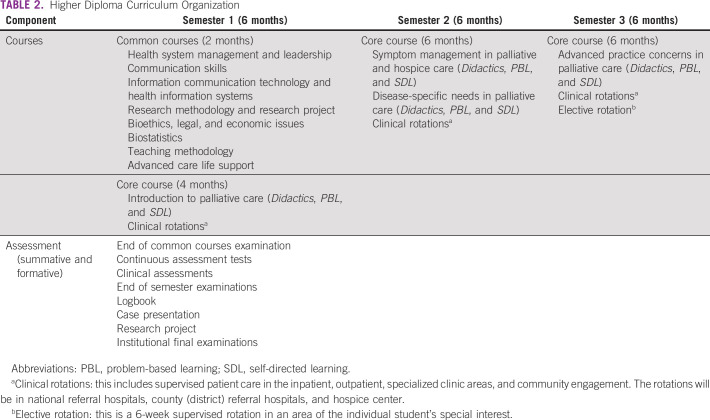
Higher Diploma Curriculum Organization

**TABLE 3:**
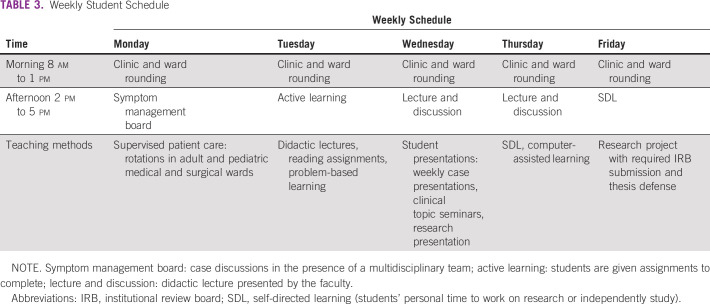
Weekly Student Schedule

## RESULTS

Misconceptions about palliative care were something we encountered frequently, with individuals believing it was predominantly pain management of cancer patients at the end of life. It was clear that building consensus and educating policymakers, regulatory boards, and government personnel at the early stages of program development was important.

Our program has enrolled seven students in its first class. Admission requirements included graduation from a CO-accredited school and a minimum of 2 years of clinical experience after graduation. The program blends much of the content and goals of North American Palliative Medicine fellowships with important requirements mandated by the Kenyan CO Council including students to develop and implement a research project during training. Our stakeholders also pointed out that being the first class of specialists in Kenya, the graduates must be prepared for administrative or educational positions. Therefore, students must complete required courses in Information Communication Technology and Health Information Systems, Health System Management and Leadership, and Teaching Methodology.

## DISCUSSION

The major challenge going forward is financial. We believe by embedding the training in a center for higher education the program can be sustained through tuition, yet there are issues. Students entering higher diploma courses are required to pay the course tuition. Many health care workers are employed by the government, and traditionally, they were permitted to attend college and retain their salary with the expectation of returning to the position with an advanced degree. Since the Kenyan government moved health care administration from the federal to the county level (devolution), the release for higher education activities is less clear and we have found many eligible students struggle to be released. We have offset the cost of tuition through partial scholarships made available through philanthropic support, but in the future, students will need to cover the tuition which may be a deterrent to program sustainability. It remains to be seen whether the first Kenyan National Palliative Care Policy, launched in October 2021, will be embraced by county governments and lead to more students being released for training or will be viewed as an unfunded mandate by the national government.

The need for palliative care is well-documented. We have learned that creating a robust, sustainable training program requires a diverse group of stakeholders with expertise in leadership, education, and the variety of clinical competencies that comprise specialty-level palliative medicine—tailored to the needs of the Kenyan people. On the basis of our experience creating a training program for COs, future directions include developing specialized training programs for nurses and physicians.
